# Influence of geographical factors on thermal stress in northern Carpathians

**DOI:** 10.1007/s00484-020-02011-x

**Published:** 2020-09-03

**Authors:** Błażejczyk Krzysztof, Nejedlik Pavol, Skrynyk Oleh, Halaś Agnieszka, Skrynyk Olesya, Błażejczyk Anna, Mikulova Katarina

**Affiliations:** 1 Climate Impacts Laboratory, Institute of Geography and Spatial Organization, Polish Academy of Sciences, Twarda 51/55, 00-818 Warszawa, Poland; 2 Earth Science Institute of Slovak Academy of Science, Dubravska cesta 9, 84005 Bratislava, Slovakia; 3 Faculty of Geography and Regional Studies, University of Warsaw, Krakowskie Przedmieście 26/28, 00-927 Warszawa, Poland; 4 National University of Life and Environmental Sciences of Ukraine, Heroyiv Oborony, 15, Kyiv, Ukraine; 5 Ukrainian Hydrometeorological Institute, Nauky, 37, Kyiv, Ukraine; 6 Bioklimatologia, Laboratory of Bioclimatology and Environmental Ergonomics, Łukowska 15/55, 04-133 Warszawa, Poland; 7 Slovak Hydrometeorological Institute, Jeseniova 15, 833 15 Bratislava, Slovakia

**Keywords:** Thermal stress, Northern Carpathians, UTCI, Mountain bioclimate

## Abstract

While general features of mountain climate are well recognised, there is not many research regarded their bioclimatic differentiation. The aim of the present study is to answer the question how different geographical factors: elevation above sea level, physiographical type of area, climate continentality and location of area in relation to the main mountain ridge influence thermal stress in northern Carpathians. To analyse thermal stress in the region, daily meteorological data from 21 stations of national weather networks of Poland, Ukraine and Slovakia for the period 1986–2015 were used. Daily data of air temperature, relative humidity, total cloud cover and wind speed at 10 m above ground for 12 UTC were used because they represent midday hours which are mostly used for any human activity. The Universal Thermal Climate Index (UTCI) was applied as a measure of thermal stress. The results show that (1) cold stress significantly increases and heat stress decreases due to rise of altitude, (2) due to climate continentality and physiographical differences between western and eastern parts of northern Carpathians in their eastern edge, the cold stress is more evident than in western one, (3) at southward slopes of Carpathian, heat stress is significantly more frequent then at northward areas.

## Introduction

Thermal stress caused by meteorological conditions plays important role in different kinds of human activity, e.g. outdoor occupation, sport, tourism and health prophylactic. Mountain regions are very sensitive areas because of wide list of factors which influence the actual weather conditions. Very important are both, general features of climate and its vertical zonation due to elevation above sea level. Local weather is also influenced by exposition of ridges and slopes to predominated winds depended on regional and local circulation patterns.

Throughout the last century, there has been conducted an active research regarding how to define bioclimatic conditions. A large number of indices have been proposed, which are (or were) in use throughout the world. The most frequently used indices were listed and discuss by Epstein and Moran (2006), Błażejczyk et al. (2012) and de Freitas and Grigorieva (2017). In the last decade, new developed Universal Thermal Climate Index (UTCI) is more and more frequently applied in bioclimatic research (Błażejczyk et al. 2012; Bröde et al. 2012; Fiala et al. 2012; Jendritzky et al. 2012; Błażejczyk and Błażejczyk 2014; Błażejczyk and Vinogradova 2014; Urban and Kyselý 2014; Błażejczyk et al. 2015; Pappenberger et al. 2015; Roshan et al. 2018).

In bioclimatic research, very important is not only the assessment of actual conditions but also explanations how and why they are changing temporally and spatially. Mountains influence climate not only of given areas but also in their surroundings. They constitute a significant physical barrier for moving air masses affecting all meteorological variables: temperature, precipitation, cloudiness, insolation etc. (Trepińska 2002; Migała 2005). It is an effect of air cooling during crossing mountain ridge which generate formation of clouds and dynamic changes in air pressure and air temperature in surrounded areas (Szmyd 2016). Important factors that affect the mountainous climate are geographical position and the orientation of the mountain ridges (Smith 2015). Lower temperature and higher wind speeds, giving a ‘wind-chill’ effect, causes that the human body is exposed to stronger thermal stress in those areas (Błażejczyk and Sitek 2003; Błażejczyk and Skrynyk 2019). In addition, in the mountains, atmospheric conditions affecting humans can significantly differ over a relatively short horizontal distance (Błażejczyk et al. 2013). Due to elevation above sea level, mountains are source of several modifications of various meteorological elements. Hitting the mountain barrier, the air masses are forced to rise which causes the cooling and the relative humidity is increasing. This causes the formation of both convective and wave clouds and results in increasing precipitation (Sturman et al. 1999; Matzarakis and Katsoulis 2006; Chena et al. 2013; Sindosi et al. 2015; Łupikasza and Niedźwiedź 2016; Kholiavchuk and Cebulska 2019; Napoli et al. 2019). Solar radiation and insolation increase due to altitude (Baranowski 2003; Żmudzka and Kulesza 2019). Nevertheless, due to the broken topography, a part of the terrain is overshadowed and the sunshine duration is limited at such places. General decrease of the temperature with rising elevation (Sturman et al. 1999; Niedźwiedź 2003, 2006; Żmudzka 2009, 2011) can be disrupted at such places and the cool air mass sitting in the deep valleys on the north site of the ridges creates frequent inversion. The wind speed is rising at elevated slopes and summits (Baranowski 1999; Błażejczyk 2019) but during the clear days, a system of permanent mountain-valley breezes of relatively low speed is formed (Wagner 1932). On the other hand, mountain passes oriented in the wind direction contribute to increased wind speed and the accumulation of the cold air behind the mountain ridge and its incursion through the mountain saddlebacks brings extreme wind speeds over 200 km/h. Altitudinal changes of meteorological variables lead to vertical diversity of ecosystems (Guo et al. 2013; Zhao et al. 2019) and heat stress in humans (Ohashi et al. 2014). Complex climatic characteristics of central European mountain ridges, including Carpathians, are done by Konček (1974), Niedźwiedź (2012), Cheval et al. (2014), Spinoni et al. (2014), Dąbrowska and Guzik (2015), Błażejczyk (2019). Some research underline the role of continentality in climate features in transitional areas both, in lowland and mountain regions (Ciaranek 2014; Vilček et al. 2016).

Until now, there are only few papers presenting biometeorological specificity of mountain areas in Europe. For example, Gajic-Čapka and Zaninović (1997) reported possible impact of temperature extremes on human perception in SE Alps, Mateeva and Filipov (2003) studied bioclimatic differentiation of Rila-and-Rhodopy, Błażejczyk and Sitek (2003) and Błażejczyk et al. (2013) discussed how elevation impact subjective temperature in Tatry Mts. Spatial and seasonal distribution of bioclimatic indices in Styria (southern Austria) was studied by Harlfinger et al. (2004), in Sudety Mts.—by Milewski (2013) and Miszuk (2008), in Croatian and Slovenian mountains—by Zaninović et al. (2006), in Mt. Zlatibor (Serbia) by Pecelj et al. (2017), and in various parts of northern Carpathians by Nowosad et al. (2013) and Bokwa et al. (2019). However, Endler et al. (2010) verified how vertical gradient of climate change influence tourism conditions in the Black Forest.

Carpathians are a wide crescent-shaped mountain ridge located in Central and Eastern Europe. It is one of the longest mountain systems in Europe (after Urals and Scandinavian Mts.) extending in arc shape for approximately 1300 km from the Danube gorge near Bratislava to the Iron Gate—the Danube gorge near Orshova. Because of its shape, it is called in geomorphology as Carpathian Arc (Kondracki 1989). Taking into account orography, the Carpathians are divided into Western, Eastern and Southern Carpathians and due to geological structure—into Outer Carpathians (composed of flysch) and Inner Carpathians (built mainly of limestone crystalline rocks) (Rączkowska et al. 2012). Central Western Carpathians (characterised by typical alpine relief) are the highest part of mountains with Tatra massif which consists of more than 50 peaks with the elevation above 2000 m (the culmination is Gerlachovský štít, 2655 m above sea level, ALT). In the Eastern Carpathians, Chornohora is the highest ridge with 6 peaks > 2000 m (Hoverla, ALT 2061 m).

In the Western Carpathians, several vertical climate zones were distinguished by Hess (1965), namely moderately warm (ALT < 700 m), moderately cool (ALT = 700–1100 m), cool (ALT = 1100–1550 m), very cool (ALT = 1550–1850 m), moderately cold (ALT = 1850–2200 m) and cold (ALT > 2200 m). For Eastern Carpathians, Niedźwiedź (2012) has proposed modified borders of those zones, respectively: ALT < 850, 850–1200, 1200–1550, 1550–1850 and 1850–2100 m (there is no cold belt). Carpathians vertical climate zones correspond with zones observed in European Alps. Rubel et al. (2017) have referred those zones to Koppen-Geiger climate classification, as follows: ALT < 1050 m—Cfb climate (coline belt), ALT = 1050–1390 m—Cfc/Dfb climate (montane belt), ALT = 1390–1880 m ALT—Dfc climate (subalpine belt), ALT = 1880–3250 m—ET climate (alpine belt).

While the knowledge of bioclimatic conditions in mountain areas is very fragmental, the aim of the present study is to answer the question how different geographical factors: elevation above sea level, physiographical type of area, climate continentality and location of area in relation to the main mountain ridge influence thermal stress in northern Carpathians.

## Materials and methods

**Fig. 1:**
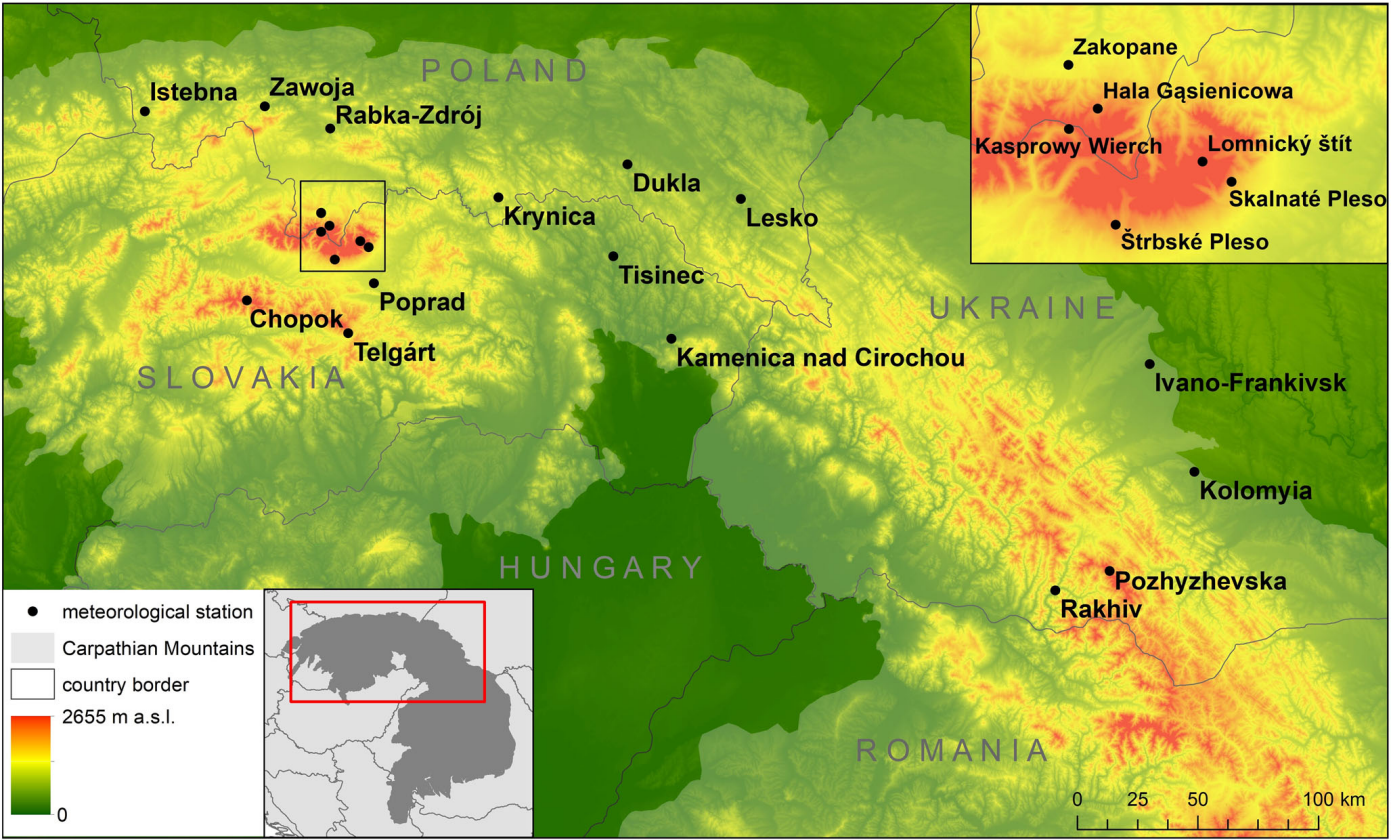
Northern Carpathians—location of meteorological stations used in research. Source: own elaboration

To analyse thermal stress in northern Carpathian region, daily meteorological data from 21 stations of national weather networks of Poland, Ukraine and Slovakia for the period 1986–2015 were used (Fig. 1). Because of its hypsometrical and physiographical differentiation, the northern Carpathians give unique opportunity to verify how various geographical factors influence bioclimatic conditions in mountain regions. In general, Polish stations represent northward slopes of Carpathian Arc and Slovak stations—its southward slopes. Ukrainian stations are located both at north-eastern as well as at south-western slopes of Carpathians Arc.

Taking into account orographic, geological and climatic facts in the present research meteorological stations were gathered in three physiographical groups: (1) coline, with elevation < 500 m and sub-mountain landscape, located mostly in Eastern Carpathians, (2) montane, with elevation of 500–1400 m and mid-mountain landscape, located mostly in Western Carpathians, (3) alpine, with elevation > 1400 m, represented mostly Central Western Carpathians with alpine relief. Considering location of station in the Carpathian Arc, the stations were classified as follows: southward, corresponded to southern and south-western slopes of the Arc, northward—located on northern and north-eastern slopes and ridges—situated on the peaks and ridges of the Arc (Table 1).

**Table 1:** Geographical information of studied meteorological stations

Name of station	Latitude	Longitude	Elevation above sea level (m)	Physiographical type	Location	*K*_G_ index (%)
Poland						
Istebna-Kubalonka (IK)	49° 36′ N	18° 54′ E	760	Montane	Northward	28.4
Zawoja (ZAW)	49° 37′ N	19° 31′ E	720	Montane	Northward	26.0
Zakopane (ZAK)	49° 17′ N	19° 57′ E	857	Montane	Northward	25.5
Hala Gąsienicowa (HG)	49° 14′ N	19° 59′ E	1520	Alpine	Northward	22.0
Kasprowy Wierch (KW)	49° 13′ N	19° 59′ E	1990	Alpine	Peak	21.0
Krynica (KRY)	49° 25′ N	20° 58′ E	595	Montane	Northward	28.3
Rabka (RAB)	49° 37′ N	19° 58′ E	510	Montane	Northward	28.0
Dukla (DUK)	49° 34′ N	21° 41′ E	360	Coline	Northward	31.0
Lesko (LES)	49° 27′ N	22° 20′ E	420	Coline	Northward	29.0
Ukraine						
Ivano-Frankivsk (IF)	48° 53′ N	24° 41″ E	275	Coline	Northward	35.5
Kolomyia (KOL)	48° 32′ N	25° 03″ E	298	Coline	Northward	36.0
Pozhyzhevska (POZ)	48° 09′ N	24° 32″ E	1451	Alpine	Northward	27.5
Rakhiv (RAK)	48° 02′ N	24° 11″ E	431	Coline	Southward	32.5
Slovakia						
Tisinec (TIS)	49° 13′ N	21° 39′ E	216	Coline	Southward	32.5
Lomnicky Štít (LS)	49° 12′ N	20° 13′ E	2635	Alpine	Peak	20.5
Skalnaté Pleso (SKP)	49° 11′ N	20° 14′ E	1778	Alpine	Southward	20.5
Štrbské Pleso (STP)	49° 07′ N	20° 04′ E	1322	Montane	Southward	25.0
Poprad (POP)	49° 04′ N	20° 15′ E	694	Montane	Southward	31.0
Chopok (CH)	48° 57′ N	19° 36′ E	2005	Alpine	Peak	22.0
Kamenica nad Cirochou (KC)	48° 56′ N	22° 00′ E	176	Coline	Southward	32.5
Telgart (TEL)	48° 51′ N	20° 11′ E	901	Montane	Southward	26.1

Source: own elaboration

For every station, daily data of air temperature, relative humidity, total cloud cover and wind speed at 10 m above ground for 12 UTC were used. The data represent midday hours which are mostly used for any human activity. The Universal Thermal Climate Index (UTCI) was applied as a measure of thermal stress (Fiala et al. 2012; Błażejczyk et al. 2012) because of its great sensibility to changes of essential meteorological variables, especially solar radiation and wind speed (Bröde et al. 2012; Psikuta et al. 2012). For the calculations of UTCI, the BioKlima©2.6 software package was used (https://www.igipz.pan.pl/bioklima.html). The Universal Thermal Climate Index (UTCI) is derived from the UTCI-Fiala model and is defined as the equivalent air temperature of reference condition causing the same model response (in sweat production, shivering, skin wettedness, skin blood flow as well as in rectal, face and mean skin temperatures) as the actual conditions (of air temperature and humidity, wind speed and mean radiant temperature). The UTCI values are categorised in 10 classes from extreme cold stress to extreme heat stress (Błażejczyk et al. 2010; Bröde et al. 2012) (Table 2). In the present research, three groups of UTCI categories are considered, namely no thermal stress (NT, UTCI = 9.1–26.0 °C), cold stress (CS, UTCI ≤ − 13 °C) and heat stress (HS, UTCI > 32 °C). The UTCI categories of moderate heat, slight cold and moderate cold stress were not considered here because of their very weak influence on physiological responses (Table 2).

**Table 2:** UTCI equivalent temperature categorised in terms of thermal stress, adapted from Błażejczyk et al. 2010; Bröde et al. 2012

UTCI (°C) range	Thermal stress category	Physiological responses
Above 46.0	Extreme heat stress (EH)	Increase in rectal temperature (*Tre*) time gradient. Steep decrease in total net heat loss. Averaged sweat rate > 650 g/h, steep increase in sweating.
38.1 to 46.0	Very strong heat stress (VSH)	Core to skin temperature gradient < 1 K (at 30 min). Increase in *Tre* at 30 min.
32.1 to 38.0	Strong heat stress (SH)	Averaged sweat rate > 200 g/h. Increase in *Tre*. Instantaneous increase in skin temperature > 0 K/min.
26.1 to 32.0	Moderate heat stress (MH)	Moderate increase in sweat rate, *Tre* and skin temperature: mean (*Tskm*), face (*Tskfc*), hand (*Tskhn*). Occurrence of sweating. Steep increase in skin wettedness.
9.1 to + 26.0	No thermal stress (NT)	Latent heat loss > 40 W. Plateau in *Tre* time gradient.
0.1 to 9.0	Slight cold stress (SLC)	Local minimum of *Tskhn* (necessary use gloves).
− 13.0 to 0.0	Moderate cold stress (MC)	Vasoconstriction. Pain due to *Tskfc* < 15 °C. Decrease in *Tskhn*. *Tre* time gradient < 0 K/h. *Tmsk* time gradient < − 1 K/h.
− 27.0 to − 13.1	Strong cold stress (SC)	Numbness due to *Tskfc* < 7 °C. *Tre* time gradient < − 0.1 K/h. Increase in core to skin temperature gradient.
− 40.0 to − 27.1	Very strong cold stress (VSC)	Frostbite due to *Tskfc* < 0 °C. Steeper decrease in *Tre*. Numbness due to *Tskfc* < 7 °C. Occurrence of shivering. *Tre* time gradient < − 0.2 K/h.
< − 40.0	Extreme cold stress (EC)	*Tre* time gradient < − 0.3 K/h. Frostbite due to *Tskfc* < 0 °C.

UTCI is expressed via climatic parameters of the particular locality. The most influential parameter in this respect is air temperature (Bröde et al. 2012) which changes both with the elevation and with the location of the station on the line sea-centre of the continent. This measure is known as climate continentality.

To assess degree of climate continentality, the Gorczyński Continentality Index (*K*_G_, %) was applied. The index has the following form (Gorczyński 1920, Ciaranek 2014):

$$ {K}_{\mathrm{G}}=1.7\ \left(\mathrm{A}/\sin\ \upvarphi \right)\hbox{--} 20.4 $$

where φ is latitude, *A* is annual temperature amplitude (°C).

Gorczyński (1920) suggests three levels of climate continentality: transitional maritime (*K*_G_, = 0–33%), continental (*K*_G_, = 34–66%) and extremely continental (*K*_G_, = 67–100%). According to Ciaranek (2014), this formula is applicable to areas in between 30 and 60° N to which our region fits.

The STATGRAPHICS Centurion XVI software package was used in statistical analysis. For verifying statistical significance of studied relations, the 95% confidence level was applied.

## Results

### General features of climate

#### Air temperature

**Fig. 2:**
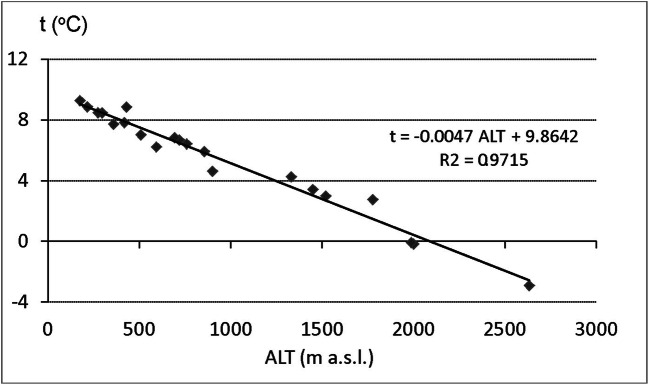
Relationships between mean yearly air temperature (*t*) and elevation above sea level (ALT), 1986–2015. Source: own elaboration

The location of the used station follows the line from IK to POZ in the direction of approximately WNW-ESE and is about 400-km long in crow-fly distance. This indicates possible changes in continental feature of the climate. Temperature range of monthly mean temperatures of warmest and coldest month rises by about 1.5 °C at comparable elevations in the line from DUK to IF and KOL. General trend of the yearly mean temperatures showed strong rise close to 1.5 °C at all stations within the period of interest but no relation between the temperature rise and elevation was recognised. The nature of the yearly course of the temperature is the same at coline and montane stations where the visible coldest month at each station is January and the warmest month is July. When moving to higher elevations at alpine stations, the coldest month is shifted to February (except for POZ) and July and August show comparable mean temperature with the differences less than 0.1 °C. Yearly mean temperatures within the region of interest balance from 8 to 9 °C in the lowest positions to 0 to − 3 °C at ALT over 2000 m. The general decrease of the temperature with rising elevation is modified by orography mostly in northerly or southerly sharply oriented high positioned valleys and slopes (KRY, SKP, RAK) (Fig. 2).

Changes in temperature comfort are on the daily basis strongly influenced by daily temperature range. Daily amplitude at alpine stations within respective 30 years is limited by 20.5 °C, and it reaches mostly 21 to 25 °C at montane stations and up to 28.5 °C at coline stations. The highest value was reached at IF and KOL which indicates the highest level of continental climate. This is also expressed in absolute temperature range which reached at IF 74.8 and at KOL 74.5 °C while it was 8–12 °C lower at stations with comparable elevation situated westward (KC, TIS, DUK) and it was limited by 55 °C at alpine stations.

#### Continentality

**Fig. 3:**
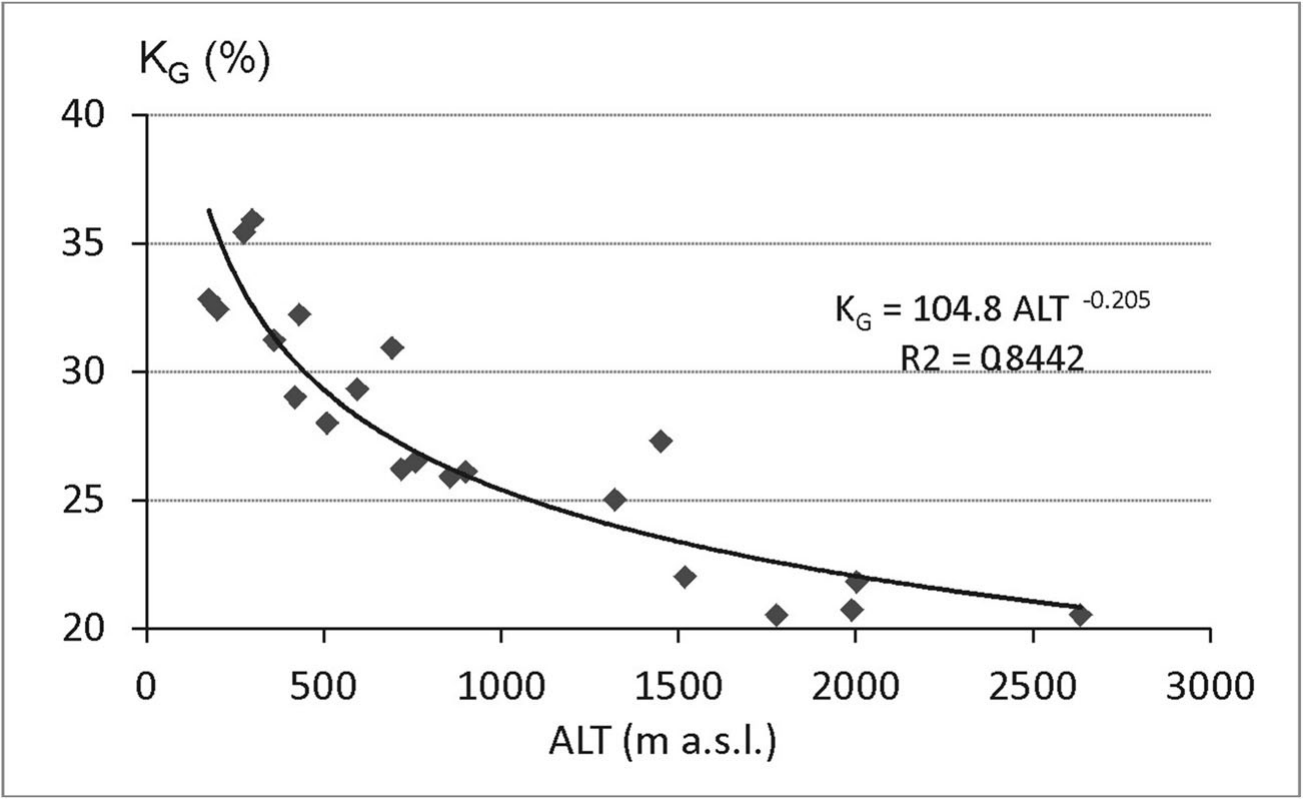
Relationships between Gorczyński continentality index (*K*_G_) and elevation above sea level (ALT), 1986–2015. Source: own elaboration

Temperature range is the most distinctive indicator of continentality. The decisive parameter in *K*_G_, annual temperature amplitude, generally raises towards the centre of the continent but decreases with the elevation. Decreasing trend of *K*_G_ according to ALT increase is seen in investigated region (Fig. 3).

Nevertheless, the changes in the direction W-E are well visible. Alpine stations located in Tatras area show the *K*_G_ from 20.5 to 22.0% but more than 350 km on the east (POZ) shows *K*_G_ = 27.3. When comparing the coline stations with similar elevation, *K*_G_ is 3–4% lower at the westerly positioned stations (DUK, TIS) than at easterly stations (IF, KOL) (Table 1). Coline stations show in general the *K*_G_ over 30% at mountain stations where *K*_G_ varies from 25 to 28%. In contrast to the strong rise of mean temperature during the respective 30 years, the *K*_G_ did not show such trend. This is in harmony found in territory of Slovakia by Vilček et al. (2016).

### General characteristic of UTCI

When analysing annual values of UTCI, one can find that most intensive heat stress occurs in RAB (northward, montane station). There is noted highest yearly UTCImean (+ 13.5 °C), UTCImax > 40 °C and frequent occurrence of HS conditions (4.6% of days yearly). Intensive heat stress in RAB is unexpected because of its relatively high elevation (ALT 510 m). This can be explained by specific location of meteorological station in the centre of warm health resort park. High UTCImean (above + 10 °C) is also observed in IK and KRY in Western Carpathians (northward, montane stations) as well as in TIS, KAM and RAK (Eastern Carpathians, southward, coline stations). Maximum UTCI > 40 °C was mostly registered at stations located in Eastern part of area (TIS, KAM, IF, RAK, KOL). Lowest values of UTCI, both mean (< − 11 °C) and minimum (< − 60 °C), are observed at most elevated Alpine stations in Western Carpathians (KW, CH, LS). Cold stress days (> 45% yearly) are very frequent at all elevated Alpine stations (including POZ). However, at stations located > 1500 m above sea level, there are no noted days with heat stress. On the contrary, HS days were registered more frequently (> 5% days per year) in stations situated at southward slopes of Eastern Carpathians (TIS, KAM, RAK) (Table 3).

**Table 3:** Mean, maximum (max) and minimum (min) annual UTCI values as well as yearly frequency of selected UTCI categories, 1986–2015

Station	UTCI values (°C)			Frequency of UTCI categories (%)		
	Min	Mean	Max	CS	NT	HS
IK	− 36.3	10.2	38.0	3.5	41.6	1.5
ZAW	− 35.3	8.5	40.1	6.6	42.1	1.5
ZAK	− 28.2	8.4	37.2	3.6	43.9	0.6
HG	− 52.1	0.3	30.1	17.5	28.0	.
KW	− 60.8	− 12.1	26.8	45.7	11.4	.
KRY	− 49.9	10.3	36.9	5.0	41.5	1.9
RAB	− 31.2	13.5	40.5	1.4	44.2	4.6
DUK	− 54.9	5.3	38.7	14.7	35.3	1.9
LES	− 45.2	6.9	38.7	10.3	38.3	1.8
CH	− 71.0	− 14.8	26.8	51.8	11.6	.
STP	− 38.2	6.6	34.1	5.8	41.7	0.2
POP	− 48.1	5.7	36.6	11.3	38.0	0.7
LS	− 73.5	− 15.7	24.2	52.2	6.4	.
SKP	− 60.3	− 1.3	28.2	20.7	23.6	.
TIS	− 47.2	10.4	42.2	7.5	37.2	5.4
KAM	− 47.4	11.3	41.3	5.0	39.5	5.1
TEL	− 58.2	2.2	33.2	18.6	35.0	0.0
IF	− 42.7	7.8	41.8	11.3	38.8	3.1
KOL	− 48.2	9.1	40.8	7.9	38.8	3.9
POZ	− 66.0	− 2.7	33.7	24.1	31.8	0.0
RAK	− 36.4	12.5	41.6	2.2	43.6	5.6

Source: own elaboration

#### Annual cycle of UTCI

**Fig. 4:**
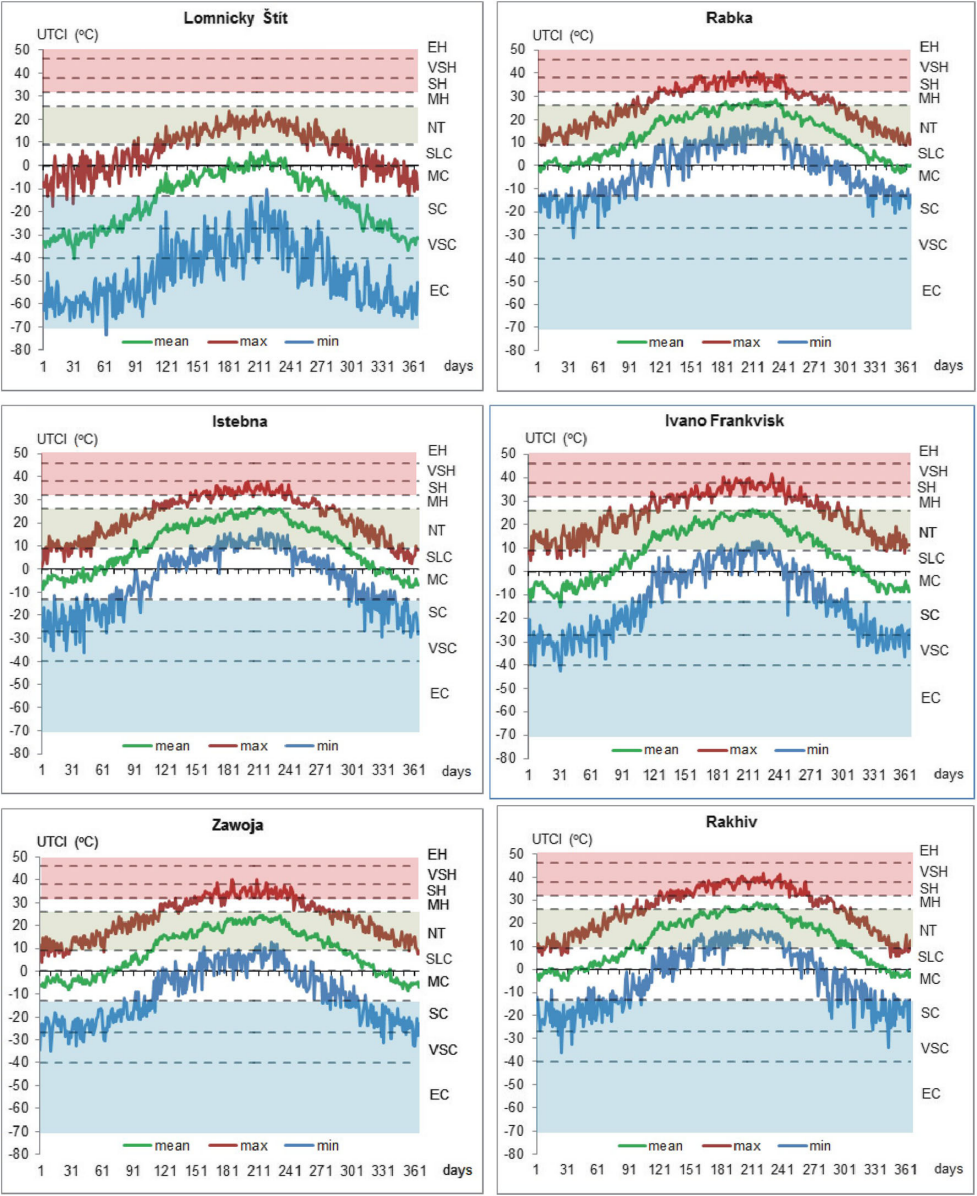
Yearly course of averaged daily UTCI values at selected meteorological stations, 1986–2015; for abbreviations, see Table 2. Source: own elaboration

Figure 4 presents averaged yearly course of mean, maximum and minimum UTCI at selected stations: coldest (LS), warmest (RAB), most western (IK), most eastern (IF), most northern (ZAW) and most southern (RAK). In general, annual course of UTCI at all stations is parallel each other. Phases of visible UTCI decreases and increases occurred at similar periods, e.g. warming phase in third decade of January and cooling periods in first decade of April and between 5 and 13 May. At similar days are also observed annual maximum of UTCI (30 July–3 August). Very evident are the lowest UTCI at LS station. UTCImax reach only no thermal stress level from middle of April until end of October. UTCImin during whole year is lower than − 13 °C and almost on every month, its value can indicate extreme cold stress. In warmest (RAB) and most southern (RAK) stations, UTCImean < 0 °C is observed only in very short periods in January, beginning of February and in December. UTCImax > 32 °C (heat stress) occurs from May until September and in summer months, its highest values exceed 38 °C. UTCImin values in winter months indicated only slight cold stress and in summer, they reach level of no thermal stress. In most eastern station (IF), summer maximums of UTCI are very similar to those observed in RAB and RAK. However, UTCImin values are very low and in winter months, they can fall below − 27 °C (very strong cold stress). Most western (IK) and most northern (ZAW) stations are located very close to each other and annual rhythm of UTCI is similar. In summer months, UTCImax reaches only level of strong heat stress. In winter season, UTCImin are usualy in the category of strong cold stress and only ocassionaly fall down to very strong cold stress level (Fig. 4).

#### Annual structure of UTCI categories

**Fig. 5:**
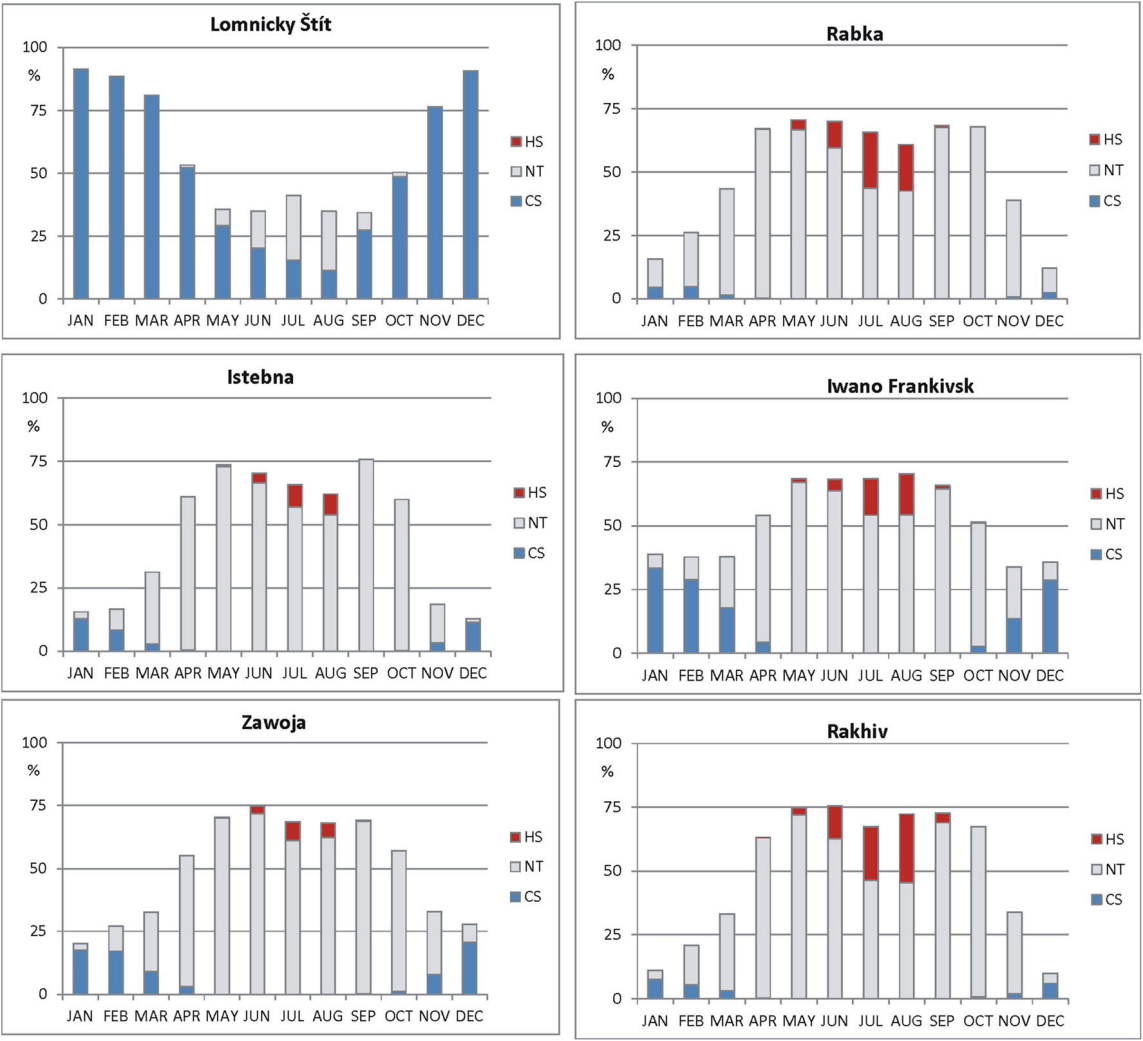
Mean Frequency of particular thermal stress categories in consecutive months, 1986–2015; HS heat stress, NT—no thermal stress, CS—cold stress. Source: own elaboration

The annual structure of particular key thermal stress categories is very differentiated in northern Carpathians. At high elevated stations (LS, CH, KW), cold stress is very frequent and from October until April, it constitutes > 50% of days monthly. No thermal stress conditions occur only from end of April until beginning of October and its highest frequency (about 10%) is observed in July. Again (similarly to annual cycles of UTCI), in the warmest (RAB) and most southern (RAK) stations, annual structure of UTCI categories is close to each other. In winter months, cold stress days are very rare (3–7%). Heat stress days are noticed from May until September and their highest frequency (> 20%) occurs in July and August. Days with no thermal stress are observed in every month with the lowest frequency in December and January (3–10%) and the highest one in spring and autumn (60–70%). In most eastern stations, both, CS and HS, days are frequent. CS days are noted from October until April with maximum occurrence in winter months (about 30%). Heat stress days are observed from May until September with the highest frequency (14–17%) in July and August. No thermal stress days in winter months are very rare (5–9%). However, from April until October, their frequency is higher than 50% (67% in May). Considering most western (IK) and most northern (ZAW) stations which are close to each other and with similar altitude, we can find that in ZAW winter, frequency of cold stress days is bigger than in IK (respectively, 17–21% to 8–13% monthly) (Fig. 5).

### UTCI and geographical factors

The above overview of thermal stress characteristics shows their spatial and seasonal differentiation in northern Carpathians. It seems that the most important factors influencing UTCI values and frequencies are elevation above sea level (compare, e.g. LS and IF stations), physiographical type of landscape (see IK and IF) and location on northward and southward slopes (e.g. RAK and IF). Thus, in this paragraph, we will discuss how the listed geographical factors influence thermal stress at studied region.

#### Elevation above sea level

In mountain areas, the altitude above sea level (ALT) is the main factor which influences air temperature. While air temperature is one of principal components of UTCI, we have verified how ALT impacts both, UTCI values and frequencies of particular UTCI categories. UTCImean significantly decreases (*r* = 0.91, *p* < 0.05) of 1.12 °C per 100-m increase of elevation. For UTCImax, altitudinal gradient is − 0.77 °C/100 m (*r* = 0.97, *p* < 0.05). However, in case of UTCImin, their values significantly (*r* = 0.59, *p* < 0.05) change according to polynomial function. At ALT below 1000 m, UTCImin increases slightly and above this elevation level, it decreases significantly. Such variability can be caused by great impact of the location of station. In low hypsometrical belt, stations are situated in various locations (valleys bottoms, elevated slopes) and every location in specific way influences air and UTCI temperature (e.g. “cold lakes” in valleys and relatively warm air elevated sites) (Fig. 3).

**Fig. 6:**
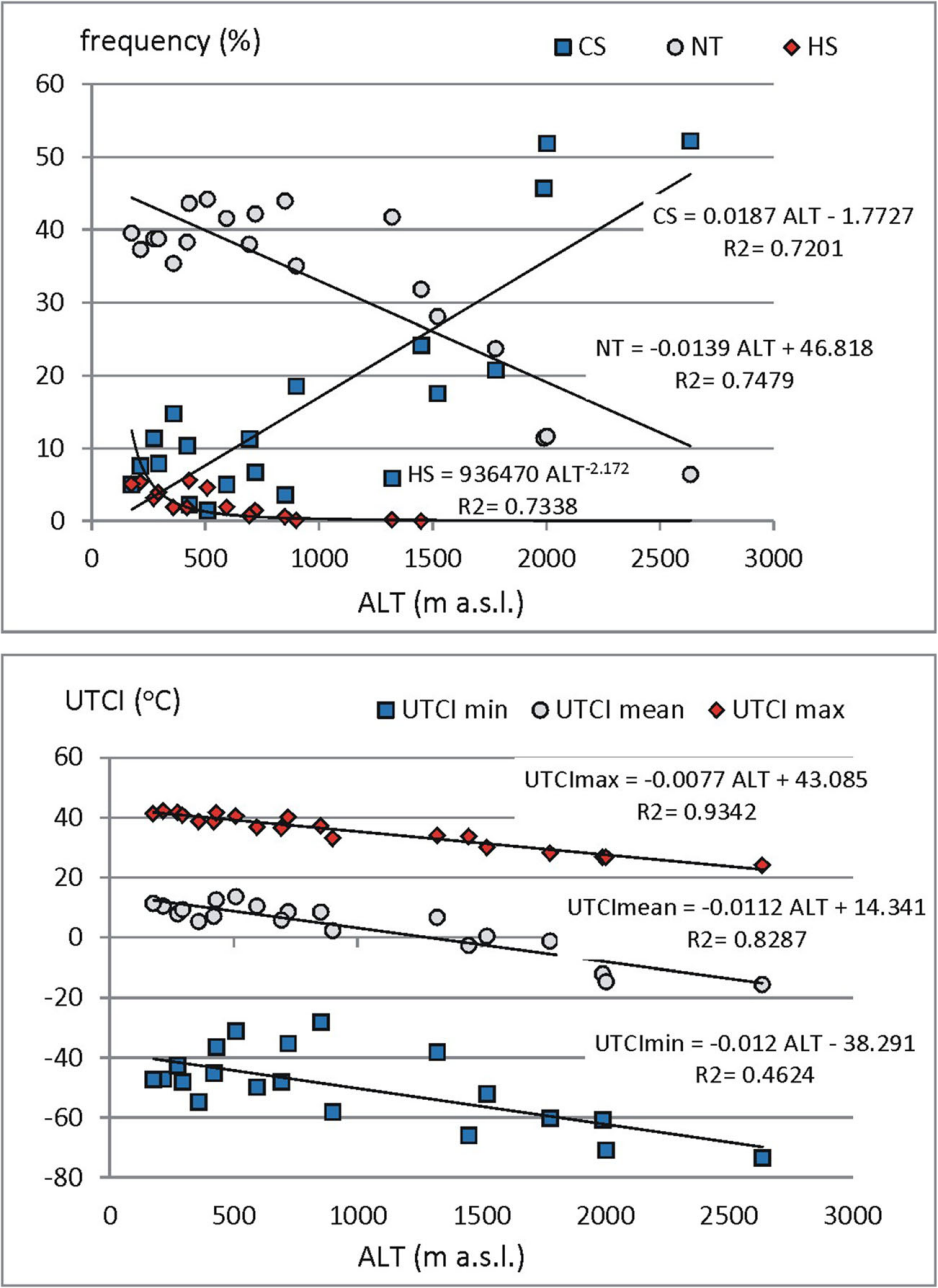
Relationships between elevation of the station above sea level (ALT) and mean yearly (UTCImean), highest (UTCImax), lowest (UTCImin) values of UTCI as well as the mean yearly frequencies of selected UTCI categories: heat stress (HS), cold stress (CS) and no thermal stress (NT). Source: own elaboration

Frequency of cold stress days (CS, UTCI < − 13 °C) rises significantly (*r* = 0.85, *p* < 0.05) of 1.9% due to altitude increase of 100 m. No thermal stress days (NT, UTCI 9–26 °C) decrease of 1.4% for every 100 m of elevation (*r* = 0.86, *p* < 0.05). However, hot stress days (HS, UTCI > 32 °C) were observed only at stations located below ALT of 1500 m. Inside this altitudinal belt, their frequency decreases significantly (*r* = 0.86, *p* < 0.05) of 0.5% per 100 m (Fig. 6).

#### Physiographical differentiation

General spatial distribution of UTCI is linked with physiographical differentiation of northern Carpathians. As previously shown, the stations at studied area were gathered into three groups: montane, coline and alpine. Alpine stations which represent areas of ALT > 1400 m are characterised by the lowest UTCI values. However, when comparing coline stations (with elevation below 500 m) and montane areas (with ALT between 500 and 1400 m), one can see that lower, eastern part of Carpathians (where coline stations are mostly located) has lower minimum UTCI values. There are also observed very high UTCI amplitudes (dUTCI=UTCImax-UTCImin) there.

Their spatial mean amplitude value is 86.7 °C which is similar to mean dUTCI for alpine stations (Table 4). Such low UTCI and high dUTCI values in coline type are probably caused by two factors: location of stations in the valleys where cold air lakes occur and increased continentality because of quite easy access—due to eastern location and absence of orographic barriers—of cold arctic and polar continental air masses. Montane stations are mostly located at elevated sites (above the range of cold lakes in valleys and basins) and open to advections of polar maritime and tropical air masses. All the discussed differences in UTCI measures between physiographical types of stations are statistically significant at *p* < 0.05.

**Table 4:** Spatially averaged values of UTCI measures and continentality index (*K*_G_) in different northern Carpathians physiographical types, 1986–2015

Northern Carpathians physiographical type	UTCI measure (°C)				*K*_G_ (%)
	UTCImin	UTCImean	UTCImax	dUTCI	
Montane	− 38.2	9.0	37.6	75.8	27.5
Coline	− 46.0	9.1	40.7	86.7	32.7
Alpine	− 60.3	− 5.7	29.1	89.4	22.3

Source: own elaboration

Significant differences between physiographical types are also seen when considering frequencies of particular UTCI categories. In alpine stations, cold stress is observed during 35% of days over the year while heat stress days do not occur at all there. In coline stations, cold stress days are more frequent (perhaps because of their location in concave forms of terrain where cold lakes of air are characteristic) then in montane areas. On the other hand, in concave forms of terrain, heat stress is more frequent than at elevated montane type areas. Both, increased occurrence of CS and HS days in coline stations (totally 12.2%) caused that NT category of thermal stress is there less frequent that in montane stations where CS and HS days constitute together only 6.9% (Table 5).

**Table 5:** Spatially averaged frequencies of UTCI thermal stress categories in different northern Carpathians physiographical types, 1986–2015

Northern Carpathians physiographical type	UTCI categories (%)		
	CS	NT	HS
Montane	5.3	41.9	1.6
Coline	8.4	38.8	3.8
Alpine	35.4	18.8	.

Source: own elaboration

#### Location

While mountains are barrier for air masses flowing across the main ridge, we have verified hypothesis that at southward (S and SW) slopes, UTCI values are higher and HS days are more frequent than at northward (N and NE) slopes of Carpathians ridge. When considering annual characteristics of UTCI, we can see that only their mean values confirm this hypothesis. At southward stations, mean yearly UTCI value is slightly higher at the level of significance of *p* < 0.05 than at northward stations. Both categories of stations also differ a little in frequency of CS and HS days. The first one are significantly (*p* < 0.05) more frequent at northward stations and the second one—at southward locations (Table 6).

**Table 6:** Spatially averaged values of UTCI measures and frequencies of UTCI categories due to various locations of northern Carpathians stations, 1986–2015

Location of station	UTCI measure (°C)			UTCI categories (%)		
	Min	Mean	Max	CS	NT	HS
Southward	− 46.3	7.5	37.3	8.8	37.3	2.8
Northward	− 44.5	7.1	37.9	9.6	38.6	1.9
Peak	− 68.4	− 14.2	25.9	49.9	9.8	.

Source: own elaboration

#### Continentality

**Fig. 7:**
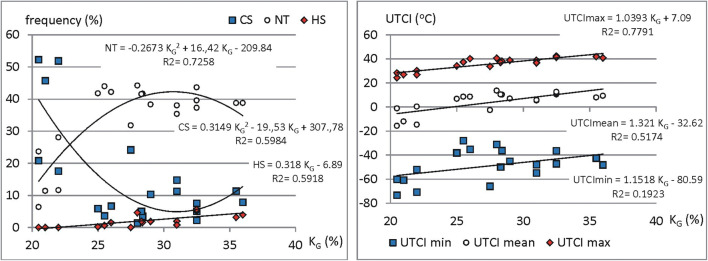
Relationships between continentality (*K*_G_) of the station and mean yearly (UTCImean), highest (UTCImax), lowest (UTCImin) values of UTCI as well as the mean yearly frequencies of selected UTCI categories: heat stress (HS), cold stress (CS) and no thermal stress (NT). Source: own elaboration

As mentioned before, the stations on the studied area differ in the degree of continentality. The values of *K*_G_ index are influenced both, by elevation above sea level and by geographical distance from the Atlantic Ocean line. While UTCI strongly depends on air temperature, there are also observed relationships between thermal stress characteristics and continentality (Fig. 7).

In general, the UTCI values rise according to *K*_G_. The best, statistically significant at *p* < 0.05 linear correlation (*r* = 0.88) was found for UTCImax. Significantly correlated with *K*_G_ are also UTCImean values. The *r* coefficient is 0.75. Insignificant are only relations between *K*_G_ and UTCImin.

In case of the frequency of days with particular UTCI categories, the number of HS days slightly increases according to *K*_G_ value. Correlation coefficient of 0.77 is statistically significant at *p* < 0.05. For CS and NT days, polynomial function of 2nd order expresses the best discussed relationships. CS days are most frequent at low KG and they rise again at high continentality index (*r* = 0.77). The contrast relationships occur for NT frequency. Low *K*_G_ generates small number of NT days. Their greatest amount is observed at *K*_G_ about 30–32% and then it fall again (*r* = 0.85).

## Discussion

Decrease of air temperature due to increase of elevation above sea level is the most important feature of mountain climate what is an effect of adiabatic cooling (Głowicki 2000; Trepińska 2002; Mateeva and Filipov 2003; Migała 2005; Cheval et al. 2014; Błażejczyk 2019; Łupikasza and Szypuła 2019). However, for particular ridges, they vary slightly because of different air humidity in particular regions and even slope exposure. For example, in Western Carpathians, Hess (1965) has found significant relationships between air temperature and elevation. Additional parameters modifying vertical thermal zonation were exposure of slopes and character of location (concave vs. convex). His findings were the base for vertical zonation of northern Carpathians and for similar research done by Niedźwiedź (2012) for Eastern Carpathians. While UTCI strongly depends on air temperature, our research also underlines vertical differentiation of thermal stress. There are only few research activities of bioclimatic conditions of mountain areas. However, they all pay attention for acceleration of cold stress or cold thermal sensation according to increase of ALT (Harlfinger et al. 2004; Zaninović et al. 2006; Miszuk 2008; Endler et al. 2010; Pecelj et al. 2017), especially at the summit zones (e.g. Błażejczyk and Sitek 2003, Błażejczyk et al. 2013).

Because of physiographical differentiation of northern Carpathians, the alpine stations are characterised by the lowest UTCI values. Coline stations (located mostly in eastern part of Carpathians) are characterised by lower mean and minimum UTCI values than montane stations (in western part of area). In coline stations, amplitude of extreme UTCI values is similar to dUTCI for alpine stations. Significant differences are also seen when considering frequencies of particular UTCI categories. It is evident that in alpine stations, cold stress is most frequent (35% of days yearly) and heat stress days do not occur there. In coline stations, both, cold stress and heat stress days, are more frequent then in montane areas.

The research shows that at southward (S and SW) slopes, UTCImean values are significantly higher and HS days are more frequent then at northward (N and NE) slopes of Carpathians ridge. CS days are significantly more frequent at northward stations and HS days—at southward locations. Similar specificity of thermal conditions due to exposure of slopes was found by Hess (1965) who defined southern slopes to be warmer then northern ones.

Physiographical character of the studied mountain area was the base of research done for Alps by Rubel et al. (2017) who have distinguished several climate belts due to their elevation and physiographical character, namely coline (< 1050 m a.s.l.), montane (1050–1390 m a.s.l.), subalpine (1390–1880 m a.s.l.) and alpine (1880–3250 m a.s.l.). Such belts correspond with different Koppen-Geiger climate zones (respectively: Cfb, Cfc/Dfb, Dfc and ET). Carpathians and Alps have similar relief patterns. However, they differ in elevation above sea level. Thus, in the present research, the Carpathian stations were gathered due to elevation and physiographical character of mountains as follows: coline—ALT < 500 m with mild slopes and sub-mountain landscape, montane—ALT 500–1400 m with sharp slopes and mountain landscape, alpine—ALT > 1400 m at elevated, open ridges and peaks. In northern Carpathians, they represent alpine type of relief and vegetation cover (Kondracki 1989, Rączkowska et al. 2012). It seems that lower limits of altitudinal belts in northern Carpathians then in Alps are related to more northern and eastern location of studied area in comparison with Alps according to Migała’s (2005) considerations.

The above UTCI evaluation was conducted for selected meteorological stations. As mentioned before, climatic conditions can significantly differ over a relatively short horizontal distance in mountain areas. More detail research by using grided data and/or some unconventional supplementary measurements within small areas (e.g. Kuba et al. 2018) can better describe the impact of local orography on bioclimatic conditions.

## Conclusions

The presented research confirms that thermal stress conditions in northern Carpathians are strongly influenced by geographical factors of that mountain massive. According to the elevation about sea level, there is observed significant decrease of UTCImean and UTCImax values with the rates of 1.12° and 0.77° per 100 m increase of elevation. Frequency of cold stress days rises significantly of 1.9%/100 m and number of no thermal stress days decrease of 1.4% per 100 m of elevation. However, heat stress days were not observed at most elevated alpine stations and at lower stations, their frequency decreases of 0.5% per 100 m.

The aim of the present study was to answer the question how different geographical factors: elevation above sea level, physiographical type of area and location of area in relation to the main mountain ridge influence thermal stress in northern Carpathians. The results of research allow to conclude that:

1. thermal stress significantly changes according to elevation above sea level: due to increase of altitude, UTCI values became lower, frequency of cold stress days increases and number of heat stress days is reduced,
2. differences in landscape physiography between western and eastern parts of Northern Carpathians cause that in coline, eastern area cold stress is more evident than in montane landscape,
3. at southward slopes of Carpathian’s arc, heat stress is significantly more frequent than at northward areas,
4. UTCI values increase due to rise of continentality and degree of continentality influences also the frequency of days with particular UTCI categories.

The next step of research should bring analysis how atmospheric circulation differentiates spatial and temporal distribution of thermal stress in mountain area of northern Carpathians.
